# Evolution of the Cp-Actin-based Motility System of Chloroplasts in Green Plants

**DOI:** 10.3389/fpls.2016.00561

**Published:** 2016-05-03

**Authors:** Noriyuki Suetsugu, Masamitsu Wada

**Affiliations:** ^1^Graduate School of Biostudies, Kyoto UniversityKyoto, Japan; ^2^Department of Biological Sciences, Graduate School of Science and Engineering, Tokyo Metropolitan UniversityTokyo, Japan

**Keywords:** blue light, chloroplast, cp-actin filament, green plants, phototropin

## Abstract

During the course of green plant evolution, numerous light responses have arisen that optimize their growth under fluctuating light conditions. The blue light receptor phototropin mediates several photomovement responses at the tissue, cellular and organelle levels. Chloroplast photorelocation movement is one such photomovement response, and is found not only in most green plants, but also in some red algae and photosynthetic stramenopiles. In general, chloroplasts move toward weak light to maximally capture photosynthetically active radiation (the chloroplast accumulation response), and they move away from strong light to avoid photodamage (the avoidance response). In land plants, chloroplast movement is dependent on specialized actin filaments, chloroplast-actin filaments (cp-actin filaments). Through molecular genetic analysis using *Arabidopsis thaliana*, many molecular factors that regulate chloroplast photorelocation were identified. In this Perspective, we discuss the evolutionary history of the molecular mechanism for chloroplast photorelocation movement in green plants in view of cp-actin filaments.

## Introduction

**FIGURE 1:**
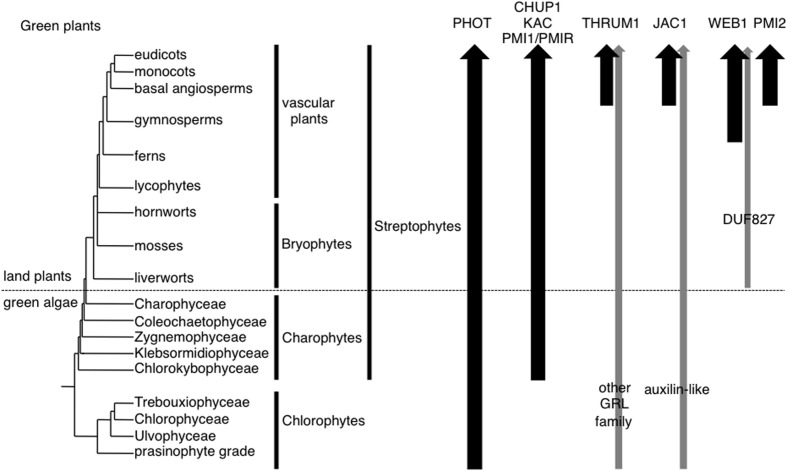
**Organismal lineages and the presence of molecular factors for chloroplast photorelocation movement.** The topology of lineages is derived from Bowman et al. (2007). Black arrows indicate the lineages in which the orthologs of respective molecular factors were identified in representatives whose genome and/or transcriptome data are available. Gray arrows indicate the lineages in which homologs of respective molecular factors were identified. Although hornworts, Chlorokybophyceae, Trebouxiophyceae, Ulvophyceae were not examined, arrows are not interrupted for clarity.

Green plants (land plants and green algae) have made many evolutionary innovations, moving from aquatic to terrestrial habitats, with multiple evolutions of multicellularity, and complex multicellular development. These evolutionary innovations also include numerous photomovement responses at the tissue, cell, and organelle levels, which allow organisms to adapt to fluctuating ambient light conditions. Chloroplast photorelocation movement (hereafter, chloroplast movement) is found in a broad range of plant and algal species including chlorophyte and charophyte green algae and land plants (Viridiplantae: Chlorophyta and Streptophyta), red algae (Rhodophyta), and photosynthetic stramenopiles (Senn, 1908). It has been extensively studied particularly in Streptophyta: embryophytes (land plants, including bryophytes, lycophytes, ferns, and seed plants) as well as Zygnemataceae (including *Mougeotia* and *Mesotaenium*) and Klebsormidiophyceae (such as *Klebsormidium*, formerly named *Hormidium;* Senn, 1908; for review, see Haupt and Scheuerlein, 1990) (**Figure 1**). Blue light is the most effective means of inducing chloroplast movement though red light is also effective in some ferns, mosses, and green algae (for review, see Suetsugu and Wada, 2007).

In most land plants, where cells have many small chloroplasts, the chloroplasts move toward weak light to capture light efficiently (the accumulation response), and they move away from strong light to reduce photodamage (the avoidance response). In general, these chloroplast movements are dependent on actin filaments in most plant species with some exceptions. In some bryopsis algae (Chlorophyta: Ulvophyceae), such as *Dichotomosiphon* and *Bryopsis*, most chloroplasts are detached from the plasma membrane and so blue light-induced chloroplast accumulation is mediated by the slowing of microtubule-dependent cytoplasmic streaming at the irradiated site, rather than by autonomous directional movement of individual chloroplasts (Mizukami and Wada, 1981; Maekawa et al., 1986). Members of the filamentous green algae *Mougeotia* and *Klebsormidium*, as well as the single-celled *Mesotaenium*, each has a single large chloroplast in a cylindrical cell. *Klebsormidium* exhibits light-induced chloroplast movement along the plasma membrane (Senn, 1908; for review, see Haupt and Scheuerlein, 1990). In *Mougeotia* and *Mesotaenium*, the ribbon-shaped chloroplast is sandwiched between vacuoles, and only the edge of the chloroplast attaches to the plasma membrane. In these algae chloroplast movement is due to the rotation of the chloroplast around the central axis of the cell (Senn, 1908; for review, see Haupt and Scheuerlein, 1990), using actin filaments (Wagner et al., 1972; Mineyuki et al., 1995). In streptophytes, microtubule-dependent chloroplast movement has only been found in the moss *Physcomitrella patens*, and this moss can use both microtubules and actin filaments for chloroplast movement (Sato et al., 2001). Thus, it is plausible that actin-dependent chloroplast photorelocation movement along the plasma membrane may have arisen subsequent to the divergence of Chlorophyta and Streptophyta (**Figure 1**) and *P. patens* evolved a microtubule-dependent system independently during land plant evolution. While algae can generally control light capture by movement in water, land plants are sessile and thus must adapt to fluctuating light conditions, such as shading by and sudden sunbeams streaming through the neighboring plants. To adapt to the harsh light conditions on land, land plants have evolved a motility system for chloroplast movement and positioning, using specialized short actin filaments around the chloroplasts, chloroplast-actin filaments (cp-actin filaments; Kadota et al., 2009; Yamashita et al., 2011; Tsuboi and Wada, 2012; Kong et al., 2013). These filaments are generated (polymerized) and employed for movement at the interface between the chloroplast and the plasma membrane (Kadota et al., 2009; Kong et al., 2013). In this Perspective, we discuss the evolution of actin-dependent chloroplast movement, and its molecular components, in green plants.

## Molecular Factors Regulating Chloroplast Movement Identified in *Arabidopsis thaliana*

**FIGURE 2:**
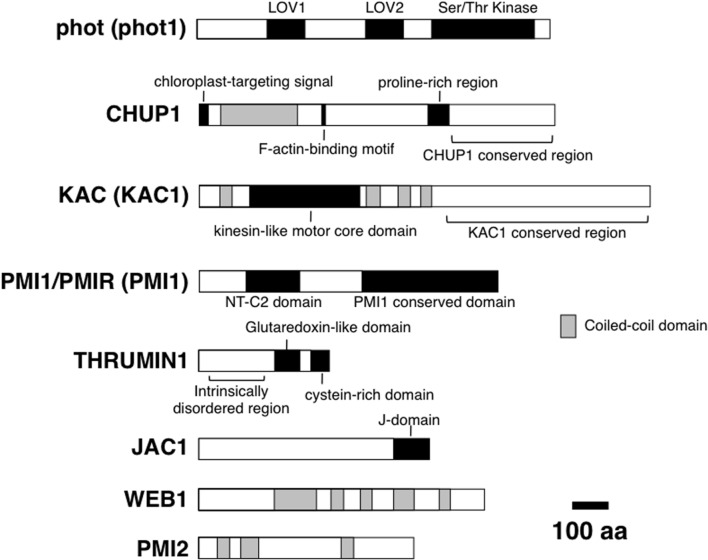
**Domain organization of molecular factors for chloroplast photorelocation movement.** For phot, KAC, PMI1/PMIR, phot1, KAC1, and PMI1 are shown. All proteins are *Arabidopsis* proteins. Functional domains (black boxes) and regions (parentheses), and coiled-coil domains (gray boxes) are shown.

Through the analysis of *Arabidopsis thaliana* mutants that are defective in chloroplast movement, many components of the photorelocation system have been identified (for review, see Wada and Suetsugu, 2013) (**Figure 2**). These include the photoreceptor kinase phototropin (phot; Jarillo et al., 2001; Kagawa et al., 2001; Sakai et al., 2001); an actin-binding protein CHLOROPLAST UNUSUAL POSITIONING 1 (CHUP1; Oikawa et al., 2003); the kinesin-like protein KINESIN-LIKE PROTEIN FOR ACTIN-BASED CHLOROPLAST MOVEMENT (KAC; Suetsugu et al., 2010); a C2 domain protein PLASTID MOVEMENT IMPAIRED 1 (PMI1; DeBlasio et al., 2005); a glutaredoxin-like protein THRUMIN 1 (THRUM1; Whippo et al., 2011); an auxilin-like protein J-DOMAIN PROTEIN REQUIRED FOR CHLOROPLAST ACCUMULATION RESPONSE 1 (JAC1; Suetsugu et al., 2005); two related coiled-coil proteins WEB1 (WEAK CHLOROPLAST MOVEMENT UNDER BLUE LIGHT 1) and PMI2 (PLASTID MOVEMENT IMPAIRED 2; Luesse et al., 2006; Kodama et al., 2010). We have demonstrated that these eight proteins (and their homologs) are essential for chloroplast movement and positioning via cp-actin filaments. Phototropins mediate chloroplast photorelocation movement via blue-light-dependent generation of cp-actin filaments (Kadota et al., 2009; Ichikawa et al., 2011; Kong et al., 2013). CHUP1 and KAC are essential for the generation of cp-actin filaments (Kadota et al., 2009; Suetsugu et al., 2010; Kong et al., 2013) and the actin-binding activity has been shown *in vitro* (Oikawa et al., 2003; Schmidt von Braun and Schleiff, 2008; Suetsugu et al., 2010). Both *chup1* and *kac* (*kac1kac2* double) mutants in *A. thaliana* are defective in chloroplast photorelocation movement and the attachment of chloroplasts to the plasma membrane (Oikawa et al., 2003, 2008; Suetsugu et al., 2010). THRUM1 is required for the efficient generation of cp-actin filaments and chloroplast movement, and co-localized with cp-actin filaments *in vivo* (Kong et al., 2013). PMI1 is required for stability of cp-actin filaments (Suetsugu et al., 2015). JAC1, WEB1, and PMI2 are involved in blue-light-induced reorganization of cp-actin filaments, although they are not essential to the generation of cp-actin filaments (Kodama et al., 2010; Ichikawa et al., 2011). These factors can be classified roughly into three categories: photoreceptor (phot), motility (CHUP1, KAC, THRUM1, PMI1), and signal transduction (JAC1, WEB1, and PMI2). This classification is based on the phenotypes in cp-actin filaments in respective mutant plants; cp-actin filaments were severely reduced or not detected in mutants deficient in factors classified as a motility category, whereas the amount of cp-actin filaments was not changed but the light-regulation was impaired in mutants deficient in factors classified as a signal transduction category. Because chloroplast movement is found universally in green algae and land plants, we subsequently investigated whether the molecular factors identified in *A. thaliana* are conserved across green algae and land plants.

## Photoreceptors and the Regulator for Motility System are Conserved in Streptophyta

The molecular factors for chloroplast photorelocation movement in green plant species other than *A. thaliana*, the fern *Adiantum capillus-veneris*, and *P. patens*, were not reported previously. As a comprehensive analysis of phototropin gene phylogeny has been already published (Li et al., 2014, 2015), we focused our search on the orthologs of CHUP1, KAC, PMI1, THRUM1, JAC1, WEB1, and PMI2. In addition to searching in NCBI GenBank’s nr database and the JGI genome database, we used the published transcriptome data from the fern *Lygodium japonicum* (Aya et al., 2015) and five charophyte algae (*Spirogyra pratensis*, *Nitella mirabilis*, *Coleochaete orbicularis*, *Klebsormidium flaccidum*, and *Mesostigma viride*; Ju et al., 2015). No orthologous proteins of these factors, including phototropins, were identified in sequence data from non-green plants, i.e., orthologs were only found in green plants. Chlorophytes do not have orthologous proteins of CHUP1, KAC, PMI1, THRUM1, JAC1, WEB1, or PMI2, but have a phototropin (Li et al., 2015). We found that there are CHUP1, KAC, and PMI1 orthologous proteins in charophyte algae as well as land plants (**Figure 1**). It was shown that CHUP1, and KAC orthologs mediated chloroplast photorelocation movement and the attachment of chloroplasts to the plasma membrane in the fern *A. capillus-veneris* and the moss *P. patens* (Suetsugu et al., 2012; Usami et al., 2012; Shen et al., 2015). In each case, cp-actin filaments were detected (Yamashita et al., 2011; Tsuboi and Wada, 2012). Furthermore, although *P. patens* uses both actin filaments and microtubules for chloroplast movements (Sato et al., 2001), CHUP1 and KAC orthologs specifically mediated the actin-dependent movements (Usami et al., 2012; Shen et al., 2015). These results suggest that the motility system using cp-actin filaments is likely to be conserved throughout land plants. *A. thaliana* has two PMI1-related proteins, PMIR1 and PMIR2: PMIR1 mediates blue light-induced plastid movement via cp-actin filaments in epidermal cells, together with PMI1 (Suetsugu et al., 2015). PMI1 and PMIR are likely to have diverged before the gymnosperm and angiosperm divergence (Suetsugu et al., 2015), suggesting that PMI1/PMIR homologs in charophyte algae and non-seed plants should be able to regulate cp-actin-filament-mediated chloroplast movement.

THRUM1 belongs to the glutaredoxin-like (GRL) protein family (of which there are 15 in *A. thaliana*); rice has the THRUM1-orthologous proteins (Navrot et al., 2006; Whippo et al., 2011). JAC1 belongs to the clathrin-uncoating factor auxilin-like proteins (of which there are seven in *A. thaliana*); rice and probably other monocot species have JAC1-orthologous proteins (Suetsugu et al., 2005). However, it remained to be determined whether these monocot *THRUM1*-like or *JAC1*-like genes are also involved in chloroplast movement. WEB1 and PMI2 belong to the DUF827 coiled-coil protein family which is divided into four subfamilies, WEB1, PMI2, WPRa, and WPRb; conifers have WEB1-orthologous proteins (Kodama et al., 2011). Although all streptophytes studied to date have GRL proteins and auxilin-like proteins (DUF827 proteins are found only in land plants), we could not identify direct orthologs of THRUM1, JAC1, WEB1, or PMI2 in charophyte algae or non-seed plants (**Figure 1**). Compared with vascular plants, the liverwort *Marchantia polymorpha* and the moss *P. patens* require much higher fluence rates of blue light to induce the avoidance response (Kadota et al., 2000; Sato et al., 2001; Komatsu et al., 2014). Our genetic analyses in *A. thaliana* suggest that WEB1 and PMI2 are necessary for suppression of the accumulation response (probably the suppression of JAC1 activity) under strong blue light (Kodama et al., 2010). Strong blue light can activate the signal transduction pathway for both the accumulation and avoidance responses (for review, see Suetsugu and Wada, 2007). Thus, the suppression of the accumulation response should be required for an efficient induction of the avoidance response, under strong light conditions. Thus, the evolution of JAC1, WEB1, and PMI2 may have permitted a more efficient avoidance response in land plants.

Thus, phot, CHUP1, KAC, and PMI1/PMIR proteins are core factors for chloroplast movement in Streptophytes. In *A. thaliana*, mutants deficient in phot, CHUP1, KAC, or PMI1/PMIR proteins exhibit severe defects in chloroplast movement and positioning (Sakai et al., 2001; Oikawa et al., 2003, 2008; Suetsugu et al., 2010, 2015) whereas mutants deficient in THRUM1, JAC1, WEB1, and PMI2 show only partial defects in chloroplast movement (Suetsugu et al., 2005; Luesse et al., 2006; Kodama et al., 2010; Whippo et al., 2011).

Although cp-actin filaments were found in *A. thaliana*, *A. capillus-veneris*, and *P. patens*, different structures of actin filaments associated with chloroplast movements were observed in some plant species using different experimental procedures (Kadota and Wada, 1989; Kandasamy and Meagher, 1999; Kumatani et al., 2006; Anielska-Mazur et al., 2009). Thus, we need to observe dynamics of actin filaments in various species using the same procedure in which cp-actin filaments were examined in *A. thaliana*. Nevertheless, the conservation of CHUP1 and KAC in streptophytes suggests that chloroplast movement in charophyte algae such as *Klebsormidium*, *Mougeotia*, and *Mesotaenium* might be regulated by cp-actin filaments. In *Mougeotia* cells fixed after irradiation with strong white light, short actin filaments have been observed at the leading edge of the moving chloroplast (Mineyuki et al., 1995), implying the presence of cp-actin filaments in *Mougeotia*. Further exploration of genetic model systems in charophyte algae will be required to elucidate the conservation across Streptophyta of the motility system for chloroplast photorelocation movement.

## Author Contributions

All authors listed, have made substantial, direct and intellectual contribution to the work, and approved it for publication.

## Conflict of Interest Statement

The authors declare that the research was conducted in the absence of any commercial or financial relationships that could be construed as a potential conflict of interest.
